# Chronicles of an uncommon term abdominal pregnancy with successful outcome: a case report

**DOI:** 10.1186/s13256-023-04312-2

**Published:** 2024-01-24

**Authors:** Joad Magezi, Joshua Wacha, Pebolo Francis Pebalo

**Affiliations:** 1 Gulu University, Gulu, Uganda; 2 Gulu Regional Referral Teaching Hospital, Gulu, Uganda

**Keywords:** Intra-abdominal pregnancy, Interdisciplinary collaboration, Exploratory laparotomy, Placental location, Surgical planning, Placental management

## Abstract

**Background:**

Intra-abdominal pregnancies, while rare, present with unique diagnostic and management challenges. We present a case of a 25-year-old para 2 + 1, black African woman, who was referred from a peripheral Health Centre level IV in the Northern part of Uganda to a Regional Referral Hospital due to an abnormal presentation with easily palpable fetal parts. This case emphasizes the significance of prompt diagnosis, interdisciplinary collaboration, surgical planning, and managing the placenta in advanced intra-abdominal pregnancies.

**Case presentation:**

A 25 year African black female, para 2 + 1 upon arrival at Regional Referral Hospital, a University Teaching Hospital during a weekend, an ultrasound was sourced from a nearby private clinic revealing an extra-uterine intra-abdominal pregnancy at 38 weeks of gestation and she was promptly sent back to the University Teaching Hospital. Following this diagnosis, the patient, who hailed from a remote village over 30 km away, returned to her family for discussions. After three days, she was readmitted. Emergency laparotomy unveiled an omental gestation sac with extensive placental attachment. A live female infant was delivered successfully, placenta was left in situ. The postoperative course was uneventful, with initial concerns about inadequate breast milk flow which resolved after placental removal during the second exploratory laparotomy.

**Conclusion:**

This case highlights the uncommon occurrence of an advanced intra-abdominal pregnancies and emphasizes the importance of multi-disciplinary teamwork and placental management. The favorable outcome in the management was based on thorough assessment of the placental location, attachments and blood supply during surgery. It demonstrates the possibility of reduced risk of massive bleeding if there is a delayed removal of placenta with favorable attachment. This is particularly important for milk letdown as well as reducing the needs of blood transfusion in resource limited settings.

## Background

Extrauterine intra-abdominal pregnancy is incredibly rare form of ectopic pregnancy where implantation is on the peritoneum, outside the uterus, fallopian tubes and or ovaries. Abdominal pregnancy accounts for 1.4% of all forms of ectopic pregnancies [1] although higher incidences have been reported in low income countries blamed on high prevalence of pelvic infection [2]. Intra-abdominal pregnancy can be classified as primary type where fertilization and implantation is in the peritoneum or secondary where fertilization occurs normally and implantation takes place in the peritoneal cavity due to majorly tubal abortion [1].

The diagnosis of abdominal pregnancy is done basing on high index of suspicion and can be confirmed by an ultrasound scan or magnetic resonance imaging [3]. The standard treatment for an abdominal pregnancy is an emergency laparotomy. In most cases, the placenta is left in place, and methotrexate is used to minimize the risk of significant bleeding [4].

Different treatment approaches have been delineated for managing the placenta post-delivery, with options spanning from complete extraction, partial removal, or leaving the placenta in its original location. The choice of treatment is contingent upon the specific site of placental attachment and involvement of major organs [5]. In this case, we intend to discuss the importance of surgical planning and placental management for an advanced term intra-abdominal pregnancy in a resource limited setting.

## Case presentation

A 25-year-old African black woman, para 2 + 1, with an uneventful antenatal history, presented with worsening abdominal discomfort during her third pregnancy in a peripheral health center level IV in remote rural area in Northern Uganda. Her last normal menstrual period was on 28th November 2022, and she was estimated to be at 34 weeks of gestation. She had received 4 previous antenatal care contact with normal findings during each visit.

In her fifth antenatal contact, upon recognition of an abnormal presentation, lie and easily palpable foetal parts at the health center, she was referred to the Regional Referral/University Teaching Hospital, for specialist management. While at referral site, she was noted to have a documented ultra-sound scan report from a private health facility at 25 weeks revealing an intrauterine pregnancy with a low-lying placenta. Because she presented late in the evening of a weekend day, the attending midwife sent her to source for another ultrasound that revealed an extra-uterine pregnancy at 38 weeks with live fetus. She was promptly sent back to the Regional Referral/University Teaching Hospital and instead, the patient returned to her village, situated over 30 km away, for discussions with her parents and husband. She returned three days later and was re-admitted. At admission, her vital signs were stable (blood pressure 110/72 mmHg, pulse rate 80 beats per minute, temperature 37 °C, respiratory rate 17 breaths per minute), and physical examination revealed, normal findings. Abdominal examination revealed easily palpable fetal parts with a breech in the lower pelvic region. Pre-operative hemoglobin was 11.8 g/dl and she had three [3] units of whole blood booked.

### Treatment and surgical procedure

**Fig. 1:**
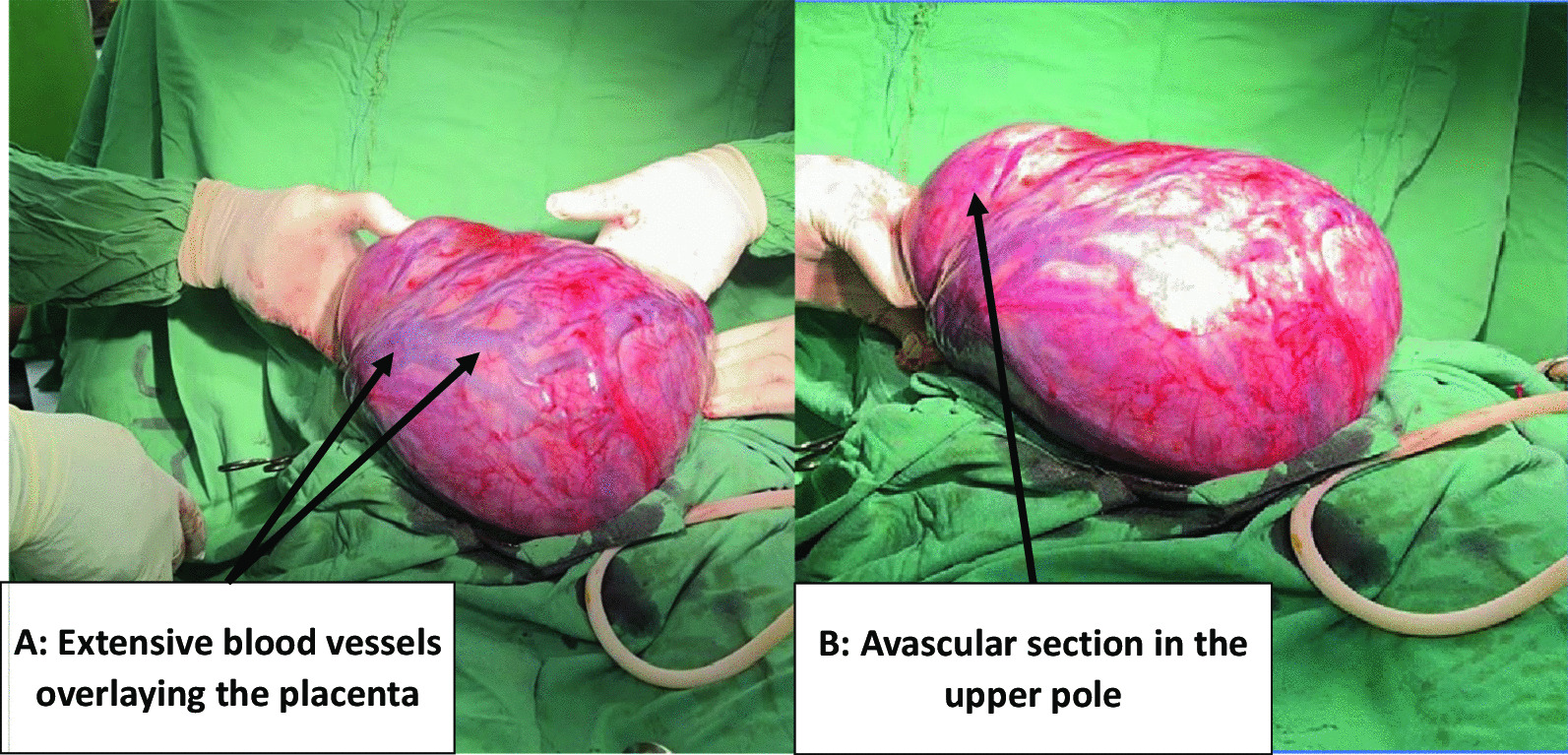
Omental gestational sac being explored. **A**-Extensive blood vessels overlaying the placenta, **B**-Avascular section in the upper pole

**Fig. 2:**
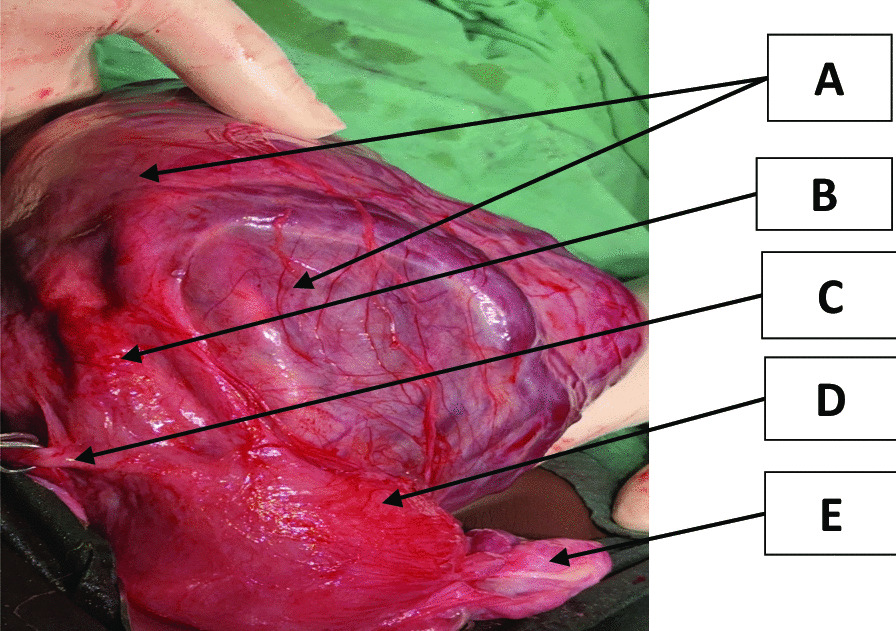
Placenta in Situ: **A**-sac containing placenta, **B**-right fallopian tube, **C**-right round ligament **D**-uterine fundus, **E**-left uterine tube and ovary

An emergency laparotomy was performed, led by obstetricians and gynecologists team involving a general surgeon (on standby), anaesthetists, and laboratory blood unit personnels. Intra-operatively, an omental gestation sac was identified completely devoid of visceral organ attachments with the placenta covering the sac’s anterior wall. Tortuous blood supply from ovarian, mesenteric, and uterine vessels were observed (Fig. 1A). The uterus appeared normal, with left fallopian tube ending blindly and the right buried at the edge of the omental gestational sac. This point to the impression of secondary abdominal ectopic according to the Studdiford Criteria [6]. The omental gestational sac was successfully delivered through an extended midline incision and explored. An incision was made on the upper less vascularized pole (Fig. 1B), less than 50mls of liquor amnii noted, and a live female baby delivered with birth weight of 2.6 kg and APGAR score of 9/10 and 10/10 at 1 and 5 minutes respectively. Umbilical cord was ligated close to the placental insertion and shortened. A gentle exploration of the placental attachment, uterus, tubes was done and documented (Fig. 2). The placenta was found to be attached within the omental sac and there was no other visceral organ involvement hence a planned remote removal was agreed on. This was to allow shrinking of the blood vessels and separation of placenta to reduce blood loss during removal. Intra-operatively, blood loss was about 300 ml which was minimalann hence there was need for transfusion.

The mother and baby were all well in the first one-week post-surgery except there was no breastmilk flows. She was planned to have the second exploratory laparotomy on day 7 post-operative but delayed to day 11 as the patient desired more consultation with her family members. The baby was reviewed by a team of pediatricians and found to be normal.

**Fig. 3:**
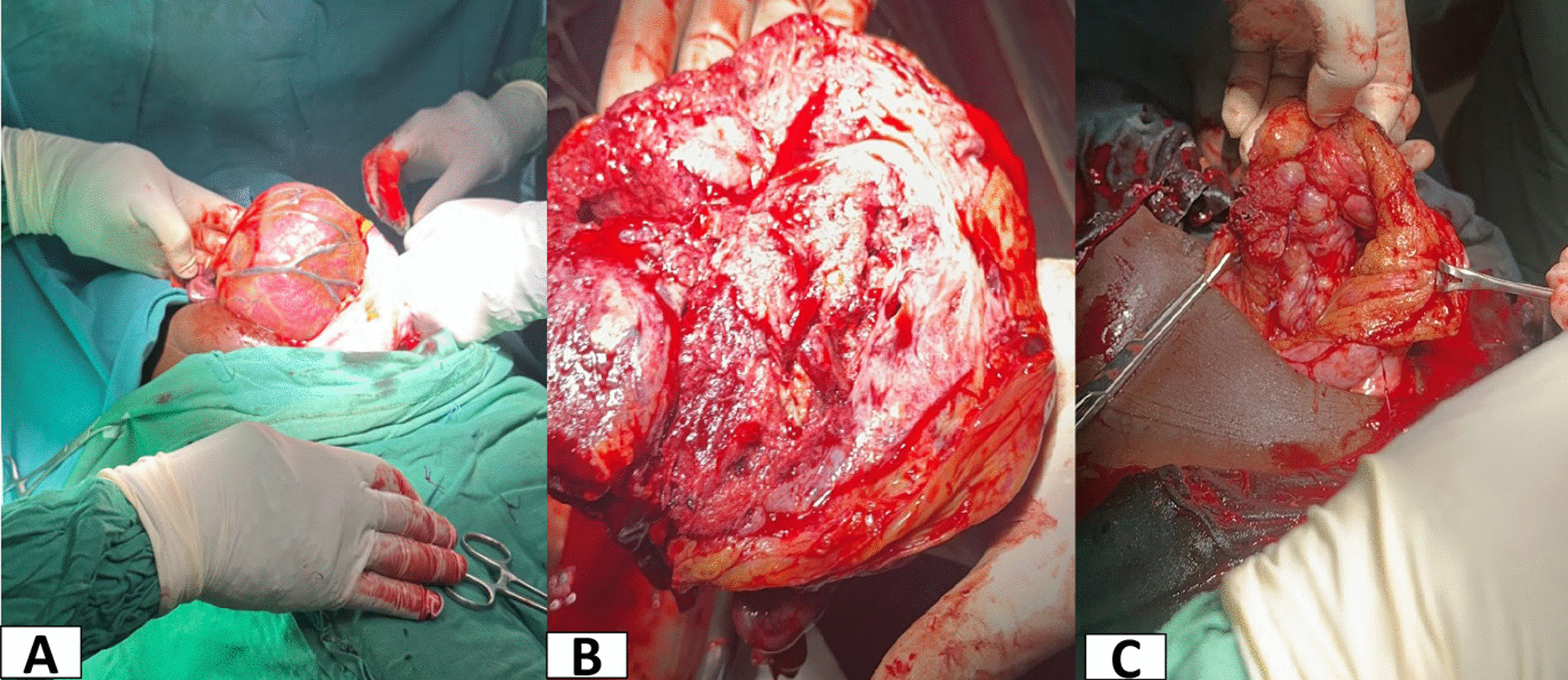
**A**-shrunken blood vessels day 11 after delivery of the Baby **B**-inner surface of the placenta after its removal; **C-**empty omental sac

Her preoperative hemoglobin before second laparopmy was 10.1 g/dl with platelet of 339,000/μl, she had stable vitals, and the previous incision wound had perfectly apposed with some serosanguinous discharge point at the peri-umbilical region. Intraoperatively, the major findings were shrunk placenta with less visible blood vessels and greyish appearance Fig. 3A). The placental bed had significant detachments with old hematoma. The placenta was delivered (Fig. 3B) and hemostasis at the bed achieved using figure of eight hemostatic sutures. The empty omental sac was closed (Fig. 3C). The estimated blood loss was 1200ml and transfused with 1 unit of whole blood intra-operatively and her hemoglobin dropped to 9.2 g/dl by third post-operative day after the second surgery. Patient was discharged on day 7 after the second exploratory laparotomy on hematinic and oral antibiotics since the incision site at the periumbilical region was slightly discharging clear non foul fluids with no swelling or induration. From the first surgery, she had no milk flow until the first post-operative day after the second laparotomy.

She returned three days later with surgical site infection that was managed successfully with daily dressings and she continued the oral antibiotics. She was reviewed at one, three and 6 weeks and all reviews were reassuring.

## Discussion

Advanced intra-abdominal pregnancies are associated with higher rate of maternal complications and death, primarily due to hemorrhage. The bleeding can arise from spontaneous rupture of the gestational sac, detachment of the placenta, or secondary to placental management during or after an intervention. In this case report, we shall emphasize the diagnostic dilemma and placental management of an advanced intraabdominal ectopic pregnancy with a live foetus in a resource-limited setting.

Misdiagnosis of intra-abdominal pregnancies is common due to the non-specific nature of their clinical presentations and difficulties in imaging either because of access or lack of requisite skills leading to a miss or delay in the diagnosis. In a case series of 5 patients presenting with advanced intra-abdominal pregnancies (gestational age 28–40 weeks) in North Eastern India, 4 had a diagnosis in the immediate pre-operative time [7]. Most patients present with suprapubic pain, bloody vaginal discharge, nausea and vomiting [8], vague abdominal tenderness and easily palpable fetal parts [9–11]. In low-resource setting antenatal clinics, mothers who present with abnormal fetal lies and easily palpable fetal parts should be a prompt for providers to evaluate for advanced intra-abdominal ectopics.

Clinically, our patient had vague adnominal pain throughout this current pregnancy compared to the previous ones and yet she had 4 antenatal contacts with all the visits documented as normal findings. In her fifth antenatal contact, the midwife in the peripheral health center realized an abnormal presentation, lie and easily palpable fetal parts and this prompted her to be referred. This case is similar to one reported by Nunyalulendho and colleague in Cameroon in which a patient presented to a nurse with an abnormal lie and easily palpable fetal parts in her fourth antenatal visit before being referred for evaluations [12].

Although the diagnosis of this intra-abdominal pregnancy was made by ultrasound scan, an earlier scan was unable to detect this. In a case series of 3 patients with intra-abdominal pregnancies in Zimbabwe, all were missed on ultrasound scans and were able to advance to term [13]. This shows the limitation of ultrasonography in the diagnosis due to skills gap [14] or otherwise. The false appearance of the placenta in our case as low-lying placentation was possible since the whole anterior-inferior parts of the omental gestation sac was covered by the placenta. Nevertheless, the second ultrasound scan was able to clearly detect this. This is similar to a case reported by Chen *et al.* in 2023 in which an intra-abdominal pregnancy was diagnosed as central placental previa [15].

Sonography has its limitations not only in missing the diagnosis but also in clearly delineating placental location which is important in planning for its management. To reduce this limitation, use of criteria such as; (1) demonstration of a foetus in a gestational sac outside the uterus; (2) failure to visualize the uterine wall between the foetus and urinary bladder; (3) proximity between the foetus and the anterior abdominal wall; and (4) localization of the placenta outside the confines of the uterine cavity has been proposed [16]. More advanced diagnostic modalities such as magnetic resonance imaging provides superior outcome compared to ultrasound scans [17] but are rarely available in resource limited-setting.

Placental management is challenging in advanced intra-abdominal pregnancies and there seems no consensus position of removal or leaving it in situ. In a large case review of 163 cases, Nunyalulendho and colleague cited that the decision to either remove or leave the placenta in situ depends on its location, attachment to retroperitoneal structures and major blood vessels [12]. Consideration of gestation age is also important in the planning for placental management as less advanced gestational age is unlikely to results into complications if managed surgically. In the case series of 13 patients with gestational age below 20 weeks, laparoscopic removal of the gestational sac was successful with normalization of serum beta-human chorionic gonadotrophins within a month [15]. In advanced cases, there are multiple approaches to placental management. A more radical approach was described in a case series of 5 patients in which 4 cases underwent total abdominal hysterectomy and one had bowel and bladder resection due to bladder and bowel attachments [7]. In a large case review of 314 patients, complete placental removal was achieved in the majority 264 (84%) of the cases meanwhile partial removal was in 27 (8.6%) and 16 cases had it left in situ [18].

Leaving the placenta in situ poses a risk of debilitating complications such as sepsis, abscess formation and delayed hemorrhages [19], and frequent follow-ups is a preferred option by some authorities. In a review of 9 cases in Tanzania, Oneko *et al.* suggested leaving the placenta as a better option due to the lack of access to whole blood in low-resource settings [20]. The use of methotrexate to aid placental autolysis has been described with contentions. It is associated with increased hospital stays due to born marrow suppressions [21] and the need for additional intervention as was reported in a review of cases in which 10 of the 18 patients on methotrexate therapy required additional surgery [18].

In our case, placental removal was deferred during the first surgery and performed during the second exploratory laparotomy when a small hematoma indicating detachment and significant evidence of placental shrinkage were observed. This approach was deemed safe and effective in reducing risks of immediate post-operative haemorrhage and requirement for massive blood transfusion and also the need for methotrexate treatment that demands extensive laboratory follow-ups. It is worth noting that removal of the placenta has been associated with immediate starts of milk let down which is key in infant survival.

Although our case had a normal live baby girl delivered with no abnormalities reported after a pediatric team assessment, it is important to note that intra-abdominal pregnancy carries several life-threatening complications to the fetus both during pregnancy and after delivery. A fetal death rate of 40–90% and 21% neonatal deformity rate has been reported [22]. Lower live birth rates of 8.6% has been reported in a case review of 314 cases [18].

The successful outcome in this case exemplifies the importance of interdisciplinary collaboration. Obstetricians, midwives and nurses, surgeons, anaesthetists, sonographers, laboratory blood unit personnel and pediatricians worked cohesively to manage the patient's condition effectively. The involvement of multiple specialties is essential for addressing the unique challenges posed by advanced intra-abdominal pregnancies.

## Conclusion

The management of advanced intra-abdominal pregnancies demands vigilance, adaptability, and a well-coordinated approach. Early and accurate diagnosis, appropriate placental management, and multidisciplinary teamwork are essential for optimizing outcomes in these cases. Delivery of placenta with favorable attachment should be done later after delivery of baby to allow spontaneous separation and regression of major placental vessels to prevent massive hemorrhage that necessitates blood transfusion, a scarce resource in many low resource settings. Delivery of placenta improves milk letdown and prevents all complications that arise when placenta is left in situ.

Although most experts advocate for termination of abdominal pregnancy, if such pregnancy present close to the viability period or the immediate pre-viability period, the possibility of continuity should always be discussed with the patients and their attendants and patients should be closely monitored. However, it is important to note that advanced intra-abdominal pregnancies carry significant risks to both the foetus and the mother.

## Data Availability

The data sets used in this case report are readily available from the corresponding author on request.
